# Exploratory Study of Andropause Syndrome in 40-65 Years in Arak: A Cross Sectional Study 

**Published:** 2018-09

**Authors:** Masomehsadat Mousavi, Mokhtar Mahmoudi, Mohamad Golitaleb, Mohammad Khajehgoodari, Davood Hekmatpou, Parya Vakilian

**Affiliations:** 1 Nursing and Midwifery Care Research Center, Faculty of Nursing and Midwifery, Isfahan University of Medical Sciences, Isfahan, Iran; 2 School of Nursing and Midwifery, Iran University of Medical Sciences, Tehran, Iran; 3 School of Nursing and Midwifery, Arak University of Medical Sciences, Arak, Iran; 4 School of Nursing and Midwifery, Tabriz University of Medical Sciences, Tabriz, Iran; 5 Department of Nursing, Faculty of Nursing, Arak University of Medical Sciences, Arak, Iran; 6 Department of Nursing, Faculty of Nursing and Midwifery, Iran University of Medical Sciences, Tehran, Iran

**Keywords:** Andropause, Testosterone, Hormone, Andropause Syndrome

## Abstract

**Objective:** Andropause syndrome is caused due to the deficiency in sex hormones and brings about symptoms of physical, psychological, and sexual nature. This study aims at investigating the prevalence of andropause syndrome in 40-65-year-old men in the central city of Iran (Arak).

**Materials and methods:** This study is a descriptive cross-sectional research conducted on 600 men living in the city of Arak in 2017. The subjects were selected through cluster sampling and qADAM was used for collecting data including three areas (level of energy, psychological and sexual). The data were analyzed through descriptive and inferential statistics (independent t-test and ANOVA) and using SPSS 16.

**Results:** The results showed that the average questionnaire score increased with an increase in age up to 50 years and above. Correlation test for the three subscales of physical, psychological, and sexual showed that the psychological subscale had the highest correlation with andropause score (p < 0.05). Among the items related to the subscales, the statement” I feel my sex drive has decreased” with mean and standard deviation of 3.62 ± 1.06 had the highest correlation with andropause and the statement “I feel I have no value for society” with mean and standard deviation of 1.7 ± 0.86 had the lowest correlation with andropause.

**Conclusion:** Andropause age in Arak is 50 years and above. Average questionnaire score showed a positive direct relation with age. Decreased sex drive had the highest correlation and losing social value had the lowest correlation with andropause state.

## Introduction

Andropause was first introduced in 1930. It means deficiency of sex hormones in men due to aging and has physical, psychological, and sexual symptoms such as fatigue, hot flashes, depression, mood changes, anxiety, loss of memory and concentration, and decreased musculoskeletal strength, which are mainly caused by a decline in testosterone level and age-related dihydrotestosterone (1). It is assumed that people with this syndrome have symptoms similar to those in women experiencing menopause (2). This syndrome in men has a slower growth than menopause in women. This syndrome was not considered important in the past, but it has gained doubled importance at present given its increase in developing countries (3). A decline in testosterone levels occurs at 1% per year after the age of 30 and the peak of this decrease is seen in men over 80, that it will be 50 percent of men at this age. The experts believe that andropause syndrome can occur at any age, but the probability of this event is approximately 20% at the age of 50 and 50%at the age of 60 (4). The elderly population in Iran has now a smaller share of the total population. However, given the fact that the country is experiencing an age structure transition from young to old as the elderly population growth rate increases by the growth of the total population and that the number and proportion of the elderly is anticipated to increase in the coming years, prospective planning necessary for controlling health-related issues emphasizes the importance of this group of the population. In 2002, UN population projections showed that assuming a moderate growth of population, in late 2040, about 18 percent of the population will be aged over 65 years (5). Population growth can cause crucial health problems for the elderly (4, 5). Along with the aging phenomenon, crucial social and economic problems will rise, one of which is andropause (6). The aging process causes changes in sex hormones levels in men, which can cause a variety of physical and psychological symptoms (7). Old age is one of the most important risk factors for developing heart failure in men. Because of old age in men, sex hormone levels (testosterone and dihydrotestosterone) strongly decrease, leading to decreased ability to exercise, exacerbation of depression, decreased libido, and susceptibility to other diseases (8). Many studies have investigated decreased testosterone levels in the elderly (1-4). In Iran, however, the only a quantitative study on andropause syndrome was conducted in 2007 by Tavoni et al. entitled as “Investigating Andropause Syndrome in 45-65-Year-Old Men in Northeast and Southwest of Tehran”. The results of this study showed that the prevalence of this syndrome was higher in men aged 55 and above and the quality of sleep and energy levels of this group decreased with an increase in age and there was a significant decrease in their sexual satisfaction and quality of marital life (9). The study of Tavoni et al. (2007) was conducted about 10 years ago and it is likely that the incidence of andopause syndrome in men has changed in terms of age and symptoms due to the changes in environmental, sociological, and nutritional factors over the time. In another study by Takizan et al. (2013) in Netherlands, the results showed that the prevalence of andropause syndrome was higher in men aged 40 to 60, there was a significant decrease in the level of hemoglobin, platelets and dihydrotestosterone in men with the syndrome, and the disorder had directly affected the quality of the subjects’ lives (10). Given the fact that many studies have shed light on the importance of this syndrome in men and of identification of its symptoms and complications (1-10) and considering the trend of population growth in Iran in recent years (11), the issue of aging and its effects on the country’s future, and the need to improve the quality of the elderly’s life on one hand and identification of this syndrome and its symptoms and complications for selecting an effective approach and improving the quality of life, the present study aimed at investigating the prevalence of andropause syndrome in 40-65 year olds in Arak.

## Materials and methods

This study was a descriptive cross-sectional research. The population for this study included the men living in Arak and with an average age of 40 to 65 years in 2017. Sample size was estimated to be 580 using $n=\frac{Z^{2}1-{\frac{\alpha }{2}}^{p(1-p)}}{d^{2}}$ and considering Z1 = a/2 = 1.96, P = 0.28, d = 0.05 and was determined to be 600 considering the sample loss of 20%. The subjects were selected through cluster sampling. First, Arak was divided into four geographical regions (North, South, East, and West) and two health centers were randomly selected from each region. Then, simple random sampling (randomly taking out number 1 or 2 out of a container) was used for selecting subjects from these two health centers. Then, the questionnaires were given to the subjects. Those who were literate and able to understand the questions, completed the questionnaire themselves and the items were read and marked for those who were unable to read. The inclusion criteria were: being 40-65, residing in Arak, having no psychological disorders (Schizophrenia and severe depression), not undergoing surgery operation in last 3 months, not taking hormone drugs, and consent to participate in this research. The exclusion criteria were: incidence of MI and being reluctant to participate in this research. The data were collected through qADAM questionnaire (quantitative Androgen decline in aging male). The first section of the questionnaire consists of personal characteristics: age, sex, marital status, level of education, number of children, length of marriage, economic condition, remarriage, smoking, addiction, BMI, pulse rate, blood pressure, history of hospitalization, current hospital stay, history of chronic diseases like diabetes, COPD, TIA, and peripheral vascular disease. The second section of the questionnaire consists of 10 items in three scales: psychological (2 questions), sexual (2 questions), and level of energy (6 questions). The qADAM questionnaire consisted the 10 questions of the original ADAM, with ‘yes’ and ‘no’ replaced by a Likert scale of 1–5, in which 5 represented the absence of a given symptom and 1 represented maximal symptoms.

All questions were weighted equally. The summation of these responses yielded a total qADAM score between 10 and 50, with 10 being most symptomatic and 50 being least symptomatic. The minimum score on this questionnaire is 14 and the maximum is 85. In terms of symptoms, scores of 14 to 49 are considered mild, scores higher than 50 moderate, and scores of 51 to 85 severe. The validity and reliability of this questionnaire has been investigated in previous studies (12, 13). This research was approved in Arak University of Medical Sciences by ethical code number IR.ARAKMU.REC.1394.353. In compliance with ethical codes, informed consent was acquired from the subjects for participating in the study and they were assured that all information collected will be confidential and will be analyzed as a group. The data were analyzed through descriptive statistics (to determine frequency, mean, and standard deviation) and inferential statistics such as independent t-test (to compare the differences in means( and analysis of variance (to determine average score of the questionnaire) using SPSS 16.

## Results

According to table 1, the majority of the participants were 45 to 50 years old (324 (50%)) and their BMI was above 25 (495 (82/5%)), It means that the majority of the participants in this research were middle-aged and were obese according to their BMI., the majority of the participants were married and had a high school degree (451 (75/2%)), were nonsmoker and not addicted, took drugs prescribed by their doctor at home (486 (81%)), had a history of hospitalization (439 (73/2%), and they had other diseases than the mentioned chronic diseases (478 (79/7)).the majority of the participants had been married for 24 years on average and had 3 children. They had stayed for about 4 days in hospital and had an average heart rate of 72/49, systolic blood pressure of 126 mmHg, and diastolic blood pressure of 79 mmHg. ANOVA test showed that the score of question naire increased with an increase in age up to 50 years (p < 0.05). This finding confirms the fact that most of the symptoms were present in people at the age of 50 and above (p < 0.05). 

**Table 1:** Personal characteristics and disease

**Variable**	**Group**		**Frequency (Per)**
Age	40-45		60(10)
	45-50		324(54)
	> 50		216(36)
Marriage	Single		0(0)
	Couple		559(93.2)
	Divorced		32(5.3)
	Widowed		9(1.5)
Education	Sub diploma		451(75.2)
	Diploma		53(8.8)
	Under master		36(6)
	Bachelor		27(4.5)
	Master & PHD		33(5.5)
Smoking	Yes		45(7.5)
	No		555(92.5)
Addiction	Yes		3(0.5)
	No		597(99.5)
Drugs	Yes		486(81)
	No		114(19)
BMI	< 19		6(1)
	19-25		99(16.5)
	> 25		495(82.5)
Athletic	Yes		284(47.3)
	No		316(52.7)
Hospitalization	Yes		439(73.2)
	No		161(26.8)
Chronic disease		COPD	3(0.5)
		Diabetes	20(3.3)
		Asthma	9(1.5)
		Heart disease	48(8)
		Stoke	39.6.5)
		Vessel disease	3(0.5)
		etc.	478(79.7)

**Table 2:** correlation index based on ADAM questionnaire sub scale

**Variable**	**Minimum**	**Maximum**	**SD ** **±min**	**Correlation**	**p-value**
Level of energy	6	22	55.3 ±66.14	0.950	0.0001
Psychologic	2	33	5.72 ± 19.40	0.980	0.0001
Sexual	2	10	1.90 ± 6.90	0.872	0.0001
Total	14	65	49.9		

Also, the average scores of the questionnaire based on the level of education had a significant difference (p < 0.05) so that the scores of the questionnaire decreased as the level of education increased. ANOVA results showed that the mean scores of the questionnaire were significantly different in a variety of chronic diseases (p < 0.05); it means that the participants were in a homogenous range of chronic diseases. The average scores of the questionnaire based on BMI levels had a statistically significant difference (p < 0.05) so that the majority of the participants were overweight (BMI > 25). Independent t-test results showed that the questionnaire scores of those smoking, using drugs, and having a history of hospitalization were significantly higher (p < 0.05) correlation test results for the three subscales of physical, psychological, and sexual showed that the psychological subscale had the highest correlation with andropause score (p < 0.05). It means that this syndrome has been more accompanied by psychological symptoms.

The results based on the table 3 showed that the highest mean and standard deviation were for the item stating “have less libido (sex drive (” with mean and standard deviation of 3.62 ± 1.06. However, in terms of correlation coefficient, the highest correlation was for the item stating “a recent deterioration in my work performance” with correlation coefficient of 0.914. The item stating “Have my lost height?” had the lowest mean and standard deviation (1.7 ± 0.86) and item stating “I am sad and/or grumpy” had the lowest correlation coefficient (353.0) (p < 0.05).

## Discussion

The results showed that the average score of the questionnaire increased with an increase in age up to 50 years, while it decreased with an increase in the level of education. It means that this syndrome is seen among low-educated people more than others. This could be due to decreased awareness of this syndrome and inability to prevent it. In a study by Tancredi in Belgium, the average age of andropause was found to be 60 (14). In Another study by Erik J in Netherlands, the average age of men with low testosterone levels and symptomatic andropause was reported to be 63 (15). In another study by Ashat et al. in India, the results were different from the rest of studies. This study reported the incidence of andropause in the age of 40-50 (16). In this study, those aged 60 to 70 mostly used herbal medicines to control their andropause, that is, they performed self-medication and the majority of the participants didn’t know of their andropause.

**Table 3:** competition of correlation index question base on physiologic, psychological, sexual sub scale

**Variable**	**Sd** **±** **min**	**MINIMUM**	**MAXIMUM**	**cORRELATION**	**p-value**
Have you noticed a recent deterioration in your ability to play sports?	0.88 ±79.1	1	4	0.82	0.0001
Are you falling asleep after dinner?	1.06 ± 3.26	1	5	0.390	0.0001
Do you have low energy?	0.98± 3.29	1	5	0.890	0.0001
Have you noticed a decreased “enjoyment in life”?	1.05 ± 3.27	1	5	0.873	0.0001
Are you sad and/or grumpy?	0.9 ± 1.81	1	4	0.353	0.0001
Has there been a recent deterioration in your work performance?	1.12 ± 3.18	1	5	0.914	0.0001
Have you lost height?	0.86± 1.7	1	4	0.477	0.0001
Have you lost weight?	1.08 ±3.23	1	5	0.905	0.0001
Are your erections not as strong?	1.03± 3.28	1	5	0.895	0.0001
Do you have less libido (sex drive)?	1.06 ± 3.62	1	5	0.695	0.0001

In this regard, this study was similar to the present study. In a study by Tkaczyszyn, the participants with andropause symptoms were 40 to 49 years old and the majority of them had a history of hospitalization and high BMI (above 25). The above studies showed that there are different results on this syndrome in men. These contradictory findings could be due to very different nutritional, behavioral, and life style factors among European and Asian countries. Also, this finding showed that the age of incidence of this syndrome in Iran, according to a similar study by Tavoni et al. was lower in Tehran, which is the most important finding of this study. Regarding the second section of the questionnaire, the correlation test results showed that the psychological subscale had the highest correlation with andropause score. It means that most people at the age of 40-50 and with andropause symptoms suffered more from psychological disorders. In a study by Yuk Yee Yan (2010) on the awareness of the andropause syndrome in middle-aged men, the results showed that most people with this syndrome had psychological disorders among which "loss of strength in getting things done" had the highest correlation with the score of andropause (17). In the studyof Tavoni et al. (2008) on investigating andropause syndrome in men in the Northeast and Northwest of Tehran, results were in conflict with those in the present study as they reported more physiological symptoms accompanied by this syndrome. In another study by Delhez (2003), andropause was found to be involved in depression and anxiety, whereas it doesn’t have any direct relationship with hormone levels (18). Psychological signs are one of the most important symptoms in this syndrome, which have a significant role in developing mental disorders in people aged 50 years and above. The difference between the results of the present study and those of similar studies (Tavoni et al.) could be due to differences in life style in North and Northeast of Tehran and Arak, which play an important role in the incidence of psychological disorders in these people In the present study, the subjects stated a decline in their sexual derive as an important result related to andropause symptoms and this disorder was mostly present at the age of 50 and above and the subjects used sexual medicine for its control. A review study by Morales (2000) to survey andropause in men showed that sexual disorders are the common problem for men with andropause syndrome. Along with these disorders, these people experience fatigue and depression as well (12). These findings are compatible with those in the present study. The study of Ashat et al. also considered sexual dysfunction associated with this syndrome as one of the most important physiological findings and concluded that people aged 60 to 70 were more likely to develop this disorder and they used herbal medicines for the treatment. In their study, Takyzan et al. found that in men aged 40 to 49 and suffering from congestive heart failure, sexual dysfunction was one of the main problems mentioned, while no discussion has been presented in relation to the controlling method in this study. In another study by Harvey on andopause in aging men, the results showed that a reduction in gonads in men with an increase in age higher than 60 was a common finding and some symptoms such as depression, fatigue, and decreased sexual function were present. Subcutaneous testosterone therapy was considered as the basic treatment for this disorder. Given the decline in sex hormones such as testosterone caused by andopause syndrome, sexual dysfunction is the common result found in the studies on this subject as well as the present study and it reconfirms the need for paying more attention to this syndrome.

## Conclusion

Given that Iran will face in the future with the population aging crisis, identifying and studying the issues and problems associated with men aged 50 and above can be effective in reducing morbidity and coping with this disorder. The results of this study indicated that andropause syndrome is occurring in people younger than the age estimated in recent studies of the syndrome and it can affect physiological, psychological, and above all, sexual abilities of the people with this syndrome. Due to the sample size and the place of conducting the present study, generalizability of the results is possible. It is recommend that further studies be done to determine the prevalence of andropause syndrome in different cities. Also, it can be studied in patients with a specific disease such as cancer. The most significant limitation of this study was shortage of financial sources to study testosterone levels in blood after reviewing the questionnaires. As this test requires more time and is expensive, it was not possible with the resources available to the present study.

## References

[1] Handelsman DJ, Liu PY (2005). Andropause: Invention, prevention, rejuvenation. Trends Endocrinol Metab.

[2] Heinemann LA, Saad F, Zimmermann T, Novak A, Myon E, BadiaX (2003). The aging male’s symptoms scales: update and complication of international versions. Health Qual Life Outcomes.

[3] Morales A, Spevack M, Emerson L, Kuzmarov I, Casey R, Black A (2007). Adding to the controversy: Pitfalls in the diagnosis of testosterone deficiency syndromes with questionnaires and biochemistry. Aging Male.

[4] Eurostat European commission (2012). Active ageing and solidarity between generations. A statistical portrait of the European Union, 2012: 73.

[5] MotihHaghshenas N (2011). Sociological aspects of aging populations and active aging challenges in Iran. Journal of Sociological Studies of Iran.

[6] Wu FC, Tajar A, Beynon JM, Pye SR, Silman AJ, Finn JD, EMAS Group (2010). Identification of late- onsethypogonadism in middle-aged and elderly men. N Engl J Med.

[7] Eurostat. European Commission Mortality and life expectancy statistics.

[8] Wong LS, van der Harst P, de Boer RA, Huzen J, van Gilst WH, van Veldhuisen DJ (2010). Aging, telomeres and heart failure. Heart Fail Rev..

[9] Taavoni S, Haghani H, PeiravieH, Jamali F (2009). andropause: experience of 45-65 years old men in north east- north west of Tehran. European Urology.

[10] Tkaczyszyn M, Nega K, Łopuszańska M, Szklarska A, Mędraś M, Ponikowska B (2013). Andropausal syndrome in men with systolic heart failure. Pol Arch Med Wewn.

[11] Yazd Kasti H, Mansori A, ZadehMohammadi A, Ahmad AbadiA (2008). The Relation of Inclination and Guilt Feeling of Divorce on Stress, Depression and Anxiety of Those Are to Divorce in Esfahan and Arak. Journal of Family Research.

[12] Morales A, Heaton JP, Carson CC 3rd (2000). Andropause: a misnomer for a trueclinical entity. J Urol.

[13] Puri S, Singh A (2015). Adam and Ams Scale for Assessing Andropause Among Aging Indian Men. Int J Pharm PharmSci.

[14] Tancred A, Reginster JY, LuyckxF, Legros JJ (2005). No major month to month variation in free testosterone levels in aging males. Minor impact on the biological diagnosis of ‘andropause,. Psychoneuroendocrinology.

[15] Duschek EJ, Gooren LJ, Netelenbos C (2005). Comparison of effects of the rise inserum testosterone by raloxifene and oral testosterone on serum insulin-likegrowth factor-1 and insulin-like growth factor binding protein-3. Maturitas.

[16] Ashat M, Puri S, Singh A, Sarpal SS, Goel NK, Koushal V (2011). Awareness ofandropause in males: a North Indian study. Indian J Med Sci.

[17] Yuk Yee Yan (2010). Awareness and Knowledge of AndropauseAmong Chinese Males in Hong Kong. Am J Mens Health.

[18] Delhez M, Hansenne M, Legros JJ (2003). Andropause and psychopathology: minor symptoms rather than pathological ones. Psychoneuroendocrinology.

[19] Harvey J, Berry JA (2009). Andropause in aging male. The Journal for Nurse Practitioners.

